# Editorial: Gene therapy 2.0: Biotechnology for circuit engineering and complex therapeutic approaches

**DOI:** 10.3389/fbioe.2022.1082883

**Published:** 2022-11-22

**Authors:** Luís Quintino, Luís Pereira de Almeida, Cecilia Lundberg

**Affiliations:** ^1^ CNS Gene Therapy Unit, Faculty of Medicine, Lund University, Lund, Sweden; ^2^ Molecular Therapy of Brain Disorders Group, Cednter for Neuroscience and Cell Biology (CNC), University of Coimbra, Coimbra, Portugal

**Keywords:** gene therapy, aav, RNA therapy, trans splicing, gene expression

The tremendous progress seen in the Gene Therapy field has been made possible by significant biotechnological breakthroughs of the last decades. In light of this, the purpose of this Research Topic is to provide a snapshot of the Gene Therapy field by assessing the status of current gene therapy technologies and if possible how close is the field to start addressing complex diseases.

The American Society for Gene and Cell Therapy defines gene therapy as “the use of genetic material to treat or prevent disease” (ASGCT). As such, there are currently two main alternatives to deliver genetic material to cells. One is to deliver therapeutic genetic material *via* non viral vectors. Alternatively, viral vectors can be used as delivery tools of therapeutic genetic payloads.

Non viral vectors have recently come to the fore with the industrious development of mRNA-based vaccines for COVID-19. It is therefore very timely that within the current Research Topic Damase et al. have written a review focusing on this type of gene therapy. The authors start briefly with the current methods employed in more conventional DNA therapeutics. Nevertheless, the bulk of the paper is a detailed description of RNA therapeutics, including the different types of therapies, an extensive assessment of current RNA therapies in clinical trials, as well as the crucial issue of delivery strategies, providing a very good entry point on this subject.

Viral vectors, on the other hand, are derived from viruses where viral particles have been reengineered to deliver therapeutic genetic material safely and efficiently. The key advantage when compared to non viral vectors is that viral vectors are substantially more effective in delivering genetic material, especially in gene therapy applications that require direct delivery to patients.

Adeno-associated viral vectors (AAV) are the viral vectors leading the way in treating rare genetic diseases in clinical trials focusing on gene replacement strategies (Mendell et al., 2020). In gene replacement approaches, viral vectors deliver a functional copy of a gene that is defective in that specific genetic disease. One key success for the field has been with the Zolgensma clinical trials (Blair, 2022). In this gene therapy clinical trial, AAV were used to successfully treat young children suffering from spinal muscular atrophy (SMA) by delivering a functional copy of the survival motor neuron 1 (SMN1) gene to cells of the brainstem and spinal cord. With clinical success stories, researchers also gain a more thorough understanding of the limitations of the gene therapy technologies used in clinical trials. For example, immunological reactions and viral vector toxicity events have been observed in non-human primates and patients (Ertl, 2022). Moreover, when expressed at very high levels certain therapeutic genes can also be toxic.

To solve the immunity and toxicity issues, researchers have turned to redesigning the AAV outer protein shell or capsid to effectively create novel artificial serotypes (Becker et al., 2022). The hope is that tailoring the AAV to the specific gene therapy application will increase transduction efficiency and decrease AAV dosages. In addition, the artificial AAV may, in theory, circumvent acquired immunity to wildtype AAVs. It is in this context that the work from Pietersz et al. provides a useful contribution. In the paper, the authors take the PHP.B peptide and assess its potential to cross the blood-brain barrier when inserted in AAV serotype 5. This peptide is a milestone in AAV capsid reengineering as it allowed a significantly more efficient brain targeting when AAV are delivered systemically (Challis et al., 2022). The authors observed that the presence of this peptide alone was not sufficient to enable blood-brain barrier crossing of the AAV5 serotype and that in the context of direct brain parenchymal striatal delivery, AAV5 seems to be a better gene therapy vehicle.

High therapeutic gene expression can be harmful, such as in inner ear gene therapy applications using neurotrophins. Peter et al. show a simple and efficient solution to this problem by combining the use of a transient viral vector with a latency promoter. With this vector and promoter combination, BDNF and NT-3 could be safely expressed, effectively protecting inner ear cells while simultaneously avoiding side-effects occurring from overexpression of these factors.

In addition to gene therapy strategies focused on gene replacement or trophic factor support as the ones highlighted above, it is possible to instead correct the gene in question by directly editing the mRNA. While RNA editing approaches such as trans-splicing has been used in several disease models, Muñiz et al. show how powerful this strategy can be. The authors used lentiviral vectors to express an RNA donor that efficiently modulates tau isoforms from 3R to 4R. They then tested this RNA editing approach in an animal model of human tau and show that shifting tau isoforms to 4R can rescue the motor and cognitive deficits observed in this model. The key strength of this study is that the therapeutic intervention was done after symptom onset, highlighting the robustness of this approach.

When taken together, the papers that are a part of this Research Topic show where the field is at. We now start to have the biotechnological tools and know-how to earnestly tackle more complex diseases. We are witnessing the “end of the beginning” of the Gene Therapy field and remain very optimistic as the field matures.
